# Increase or Decrease Hydrogen Sulfide Exert Opposite Lipolysis, but Reduce Global Insulin Resistance in High Fatty Diet Induced Obese Mice

**DOI:** 10.1371/journal.pone.0073892

**Published:** 2013-09-13

**Authors:** Bin Geng, Bo Cai, Feng Liao, Yang Zheng, Qiang Zeng, Xiaofang Fan, Yongsheng Gong, Jichun Yang, Qing hua Cui, Chaoshu Tang, Guo heng Xu

**Affiliations:** 1 Department of Physiology and Pathophysiology, School of Basic Medical Science, Peking University, Bei Jing, P.R. China; 2 Department of Physiology, Guangdong Medical Collage, Zhan Jiang, Guang dong Province, P.R. China; 3 International Medical Center, Chinese PLA General Hospital, Bei Jing, P.R. China; 4 Institute of Hypoxia Medicine, Wenzhou Medical University, Wen Zhou, Zhe jiang Province, P.R. China; Virgen Macarena University Hospital, School of Medicine, Spain

## Abstract

**Objective:**

Adipose tissue expressed endogenous cystathionine gamma lyase (CSE)/hydrogen sulfide (H_2_S) system. H_2_S precursor inhibited catecholamine stimulated lipolysis. Thus, we hypothesized that CSE/H_2_S system regulates lipolysis which contributed to the pathogenesis of insulin resistance.

**Methods:**

We treated rat adipocyte with DL-propargylglycine (PAG, a CSE inhibitor), L-cysteine (an H_2_S precursor) plus pyridoxial phosphate (co-enzyme) or the H_2_S chronic release donor GYY4137, then the glycerol level was assayed for assessing the lipolysis. Then, the effects of PAG and GYY4137 on insulin resistance in high fatty diet (HFD) induced obese mice were investigated.

**Results:**

Here, we found that PAG time-dependently increased basal or isoproterenol stimulated lipolysis. However, L-cysteine plus pyridoxial phosphate or GYY4137 significantly reduced it. PAG increased phosphorylated protein kinase A substrate, perilipin 1 and hormone sensitive lipase, but L-cysteine and GYY4137 decreased the parameters. In HFD induced obese mice, PAG increased adipose basal lipolysis, thus blunted fat mass increase, resulting in lowering insulin resistance evidenced by reduction of fasting glucose, insulin level, HOMA index, oral glucose tolerance test (OGTT) curve area and elevating the insulin tolerance test (ITT) response. GYY4137 inhibited lipolysis in vivo without increasing fat mass, but also ameliorated the insulin resistance in HFD mice.

**Conclusion:**

These results implicated that inhibition endogenous CSE/H_2_S system in adipocytes increased lipolysis by a protein kinase A-perilipin/hormone-sensitive lipase pathway, thus blunted fat mass increase and reduced insulin resistance in obese mice; giving H_2_S donor decreased lipolysis, also reduced insulin resistance induced by HFD. Our data showed that increase or decrease H_2_S induced opposite lipolysis, but had the same effect on insulin resistance. The paradoxical regulation may be resulted from different action of H_2_S on metabolic and endocrine function in adipocyte.

## Introduction

Obesity is popular diseases in developed and developing countries and major characteristic is fat mass increase. In obesity individual, un-balance of over energy uptake and lowered energy expenditure is the major reason of obesity. All mammals store excess amounts of energy in the form of intracellular triglycerides, mainly in lipid droplets. During food deprivation or stress, triglyceride lipolysis provides the primary source of energy [1]. In obese adipocyte, starvation or stress-stimulated lipolysis reduced, but basal triglyceride lipolysis elevated then released more free fatty acids (FFAs) into the bloodstream. The excess FFAs from obese adipocyte induced target tissues local inflammatory response, oxidative stress, endoplasmic reticulum stress and metabolic disorder etc. which seem to be metabolic risk factors contributing to the pathogenesis of diabetes and insulin resistance [2]. Three major lipases control lipolysis: adipose triglyceride lipase (ATGL), hormone-sensitive lipase (HSL) and monoglyceride lipase [3]. ATGL exhibits high substrate for triacylglycerol [4] and mediates basal lipolysis [5]. HSL is a well-known rate-limiting enzyme of lipolysis under starvation and stress [1]; PKA phosphorylated HSL at Ser^659^, and Ser^660^ site increased [6], and AMP-activated protein kinase (AMPK) phosphorylated HSL at Ser^565^ [7] inhibited HSL activity. Perilipin 1 (perilipin A) is a major lipid droplet scaffold protein and blocked the access of cytosolic lipases to lipid droplet. Phosphorylation perilipin by PKA results in perilipin conformational changes that expose lipid droplet stores and facilitates translocation of phosphorylated HSL, thereby elevating the fat mobilization [8].

Hydrogen sulfide (H_2_S) is a gasotransmitter and plays important regulatory roles in cardiovascular, gastrointestinal and neurological diseases [9], [10]. Cystathionine β synthase (CBS), cystathionine γ lyase (CSE) or 3-mercaptopyruvate sulfurtransferase are key enzymes generating H_2_S as L-cysteine as a substrate [11]. Our recent work found that visceral white adipose, subcutaneous adipose and perivascular adipose tissues expressed CSE protein and endogenously generated H_2_S [12], [13]. Interestingly, in normal culture condition, an H_2_S donor inhibited basal or insulin-stimulated glucose uptake in mature adipocytes,whereas blocked endogenous H_2_S production by DL-propargylglycine (PAG) increased glucose uptake activity [12]. However, in 3T3-L1 differentiated adipocytes exposed to high glucose (25 mM), H_2_S or its precursor L-cysteine increased glucose utilization [14]. These works suggested H_2_S might play different roles in glucose utilization in physiological and diabetic condition, which also means that H_2_S might regulate balance of energy storage (lipid accumulation) and consumption (lipolysis) while adipocyte is in different energy statues. H_2_S precursor-cysteine dose-dependently inhibited catecholamine-stimulated lipolysis or inhibited HSL activity with TNF-α stimulation [15], [16] in rat adipocytes. So we hypothesized that adipocyte endogenous CSE/H_2_S pathway regulated lipolysis, which contributed to insulin resistance induced by obesity.

To test our hypothesis, we used PAG inhibition of CSE activity and GYY4137 as H_2_S donor, to investigate the possible role of endogenous CSE/H2S system in adipose lipolysis. To confirm the effect in vivo, we also evaluated the effects of PAG and GYY4137 on lipolysis and insulin resistance in obesity mice induced by HFD.

## Materials and Methods

### Material

Male Sprague-Dawley rats (180–200 g) and C57BL/6J mice (13–15 g) were provided by the Animal Department, Health Science Center of Peking University. All animal care and experimental protocols complied with the Animal Management Rules of the Ministry of Health of the People’s Republic of China and the guide for the Care and Use of the Laboratory Animals of the Peking University. L-cysteine, pyridoxal phosphate, isoproterenol, bovine insulin and phenol red-free DMEM were from Sigma (St. Louis, MO). Polyclonal antibody against rat perilipin was a gift from C. Londos (US National Institutes of Health). Antibodies recognizing HSL, phosphorylated HSL (phospho-HSL), phospho-PKA substrate (RRXS*/T*), AMPK and IRS-1 were from Cell Signaling Technologies (Boston, MA). Defatted bovine serum albumin (BSA) and enhanced chemiluminescence (ECL) reagent were from Applygen Technologies (Beijing).

### Primary Adipocyte Isolation and Culture

Primary adipocytes were isolated from epididymal fat pads of male Sprague-Dawley rats (160∼180 g) according to our laboratory method [17]. The minced fat pads (2 g) were suspended in Krebs-Ringer solution (5 mL) containing 0.75 mg/ml type I collagenase, 200 nM adenosine, 25 mM Hepes, pH 7.4, and 1% free fatty acid free-BSA, then digested at 37°C by horizontal shaking (110 rpm) for 30 min. Primary adipocytes were collected, washed and counted, then pre-incubated in phenol red-free and serum-free DMEM in an atmosphere of 5% CO_2_ at 37°C for 1-h before treatments.

### Evaluation of Lipolysis by Glycerol Assay

Adipocyte lipolysis leads to the hydrolysis of triglyceride release of three free fatty acids and a glycerol backbone. Thus, we measured glycerol content in medium to assess lipolytic reaction. Adipocytes were incubated in serum-free and phenol red-free DMEM. After treatment with different reagents for different times, the culture medium was collected and heated at 70°C for 10 min to inactivate residue lipase activity. Glycerol was determined by the enzyme-coupled colorimetric assay (GPO Trinder reaction) [18]. For evaluation adipose tissues lipolysis, 20 mg adipose tissue from normal chow mice or high fatty diet mice was incubated in serum-free and phenol red-free DMEM for 1-h, the release glycerol was measured.

### Western Blot Assay

Adipose tissues or adipocytes were collected and lysed in buffer containing 62.5 mM Tris-HCl, pH 6.8, 2% SDS, 0.1 mM sodium orthovanadate, and 50 mM sodium fluoride. The lysates were centrifuged by 12000 g at 4°C, then solid lipid was removed, and protein content was determined by the BCA assay. Equal amounts of protein were denatured, then loaded and separated by SDS-PAGE. For detecting phospho-perilipin with anti-perilipin antibody, we used a low*-*Bis–concentration polyacrylamide gel prepared with 10% acrylamide and 0.07% N, N′-methylene-bis-acrylamide (at 142∶1 ratio *versus* 39∶1 for traditional gel), to provide maximal resolution of proteins in the 60- to 70-kDa range [17], [19]–[21]. Proteins transferred on membranes were recognized with use of primary antibodies and horseradish peroxidase-conjugated secondary antibodies. The bands were developed by use of ECL reagents. If required, membranes were stripped by a commercial stripping buffer (2% SDS, 62.5 mM Tris-HCl pH 6.8, 0.8% β-mercaptoethanol), and blots were reprobed with other antibodies.

### High Fatty Diet Induced Insulin Resistance Mice

Male C57BL/6J mice were housed in standard cages in a temperature- and mumidity-controlled environment, on a 12-h light/dark cycle, and with free access to water. From the age of 7-wk, animals were fed a normal diet (equivalent of 10% energy from fat) or a high-fat diet (HFD: equivalent of 45% energy from fat, both purchased from Beijing HFK Bioscience CO. LTD). After 12-wk of feeding, an oral glucose tolerance test (OGTT) and insulin tolerance test (ITT) were performed on non-anesthetized mice. Mice were fasted for 16-h before OGTT and for 4-h before ITT. For OGTT, blood (tail vein) glucose levels were measured at baseline and 15, 30, 60, 90, and 120 min after gavage (gastric tube, outer diameter 1.2 mm) glucose (150 mg) by using an Accu CheK Active glucometer (Roche Diagnostics). For ITT, the blood glucose levels were measured at baseline and 15, 30, 45, 60 and 90 min after injection bovine insulin (0.25 U/kg body weight). For HFD feeding 13-wk, mice were fasted 6-h and blood was collected by eyeball. Fasting blood glucose was determined by glucose oxidase method. Fasting serum insulin was determined radioimmunochemically using a rabbit anti-mouse insulin antibody, ^125^I-labeled bovine insulin as tracer, and mouse insulin as standard. HOMA index was counted by (fasting glucose ×fasting insulin)/22.5.

### Statistical Analysis

Data are means ± SD. Differences among groups were analyzed by one-way ANOVA, then Student-Newman-Keuls test or nonparametric *t* test. A *P*<0.05 was considered statistically significant.

## Results

### Inhibition of the Endogenous CSE/H_2_S System Increased Primary Adipocyte Lipolysis

**Figure 1:**
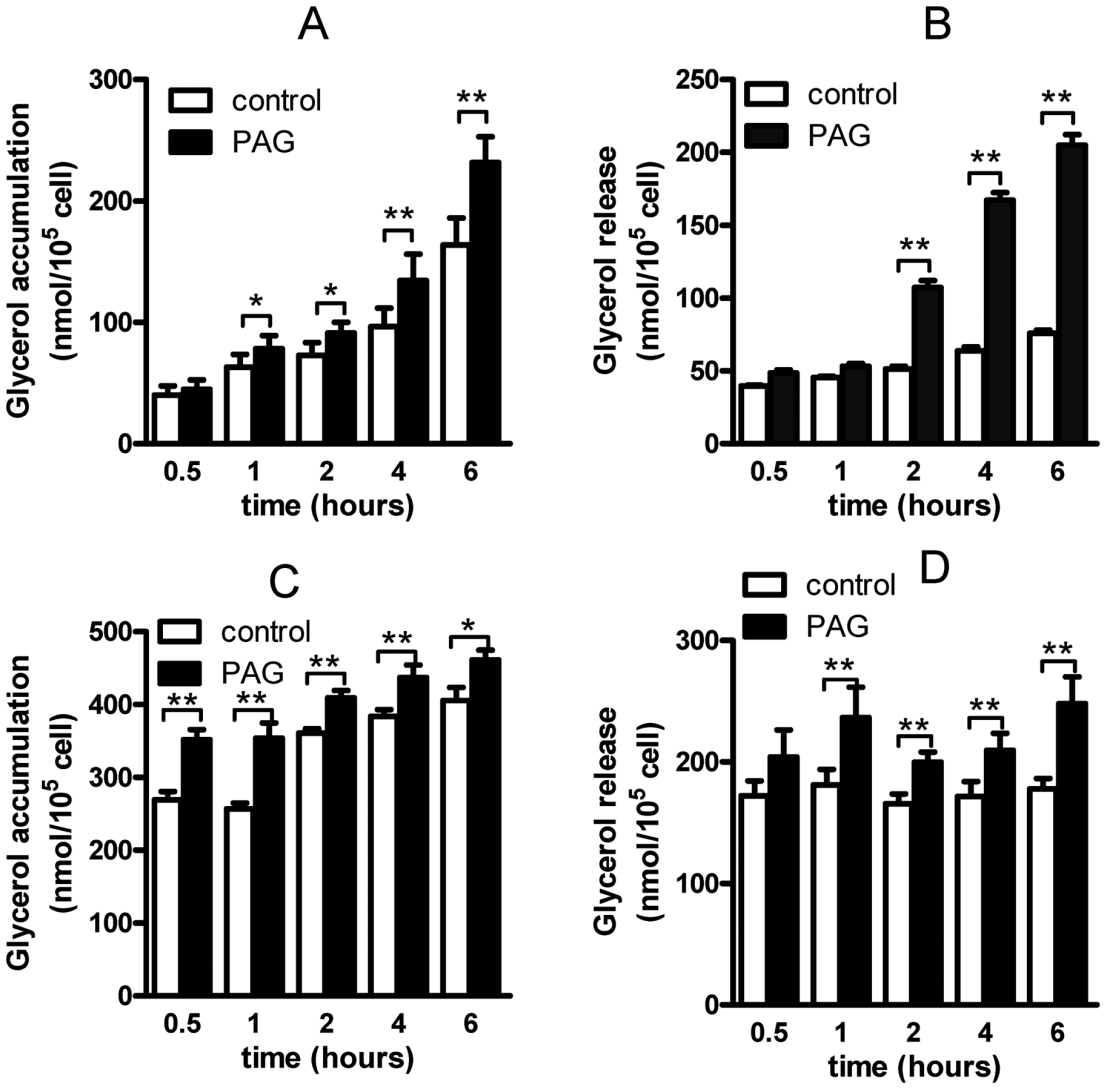
Inhibiting cystathioninine gamma lyase by DL-propargylglycine (PAG) increased lipolysis in rat adipocytes. Glycerol accumulation in isolated rat adipocytes with PAG treatment for different times (A), then medium was changed to fresh Krebs-Ringer buffer for 1 hr and glycerol release was measured (B). After PAG treatment for different times, isoproterenol (1 µmol/L) was given for 1 hr, then glycerol accumulation (C) and release (D) was assayed. Data are mean ± SD of 15 to 20 experiments. * P<0.05; **P<0.01 versus control.

Primary adipocytes only expressed CSE/H_2_S system [12], but not expressed CBS (data not shown). Here, we used PAG (200 µmol/L) to block endogenous CSE activity, which time-dependently increased glycerol accumulation in the culture medium (Fig. 1A, P<0.05). After PAG treatment, we immediately changed the culture medium to fresh Krebs-Ringer buffer, then continue to culture for one hour, the glycerol level (glycerol release) was also time-dependently increased (Fig. 1B, P<0.05). PAG treatment for 8-h or elevated PAG concentration (up to 1 mmol/L) did not increase glycerol release (data not shown), which implied that effect of PAG mainly due to blocking CSE activity. To investigate the PAG effect on lipolysis during stress condition, we observed the PAG effect on lipolysis induced by isoproterenol, and found that PAG pretreatment for different times slightly increased lipolysis response to isoproterenol (1 µmol/L, for 30 min, Fig. 1C and D). These data suggested that blocking endogenous CSE/H_2_S system stimulated adipocyte lipolysis.

**Figure 2:**
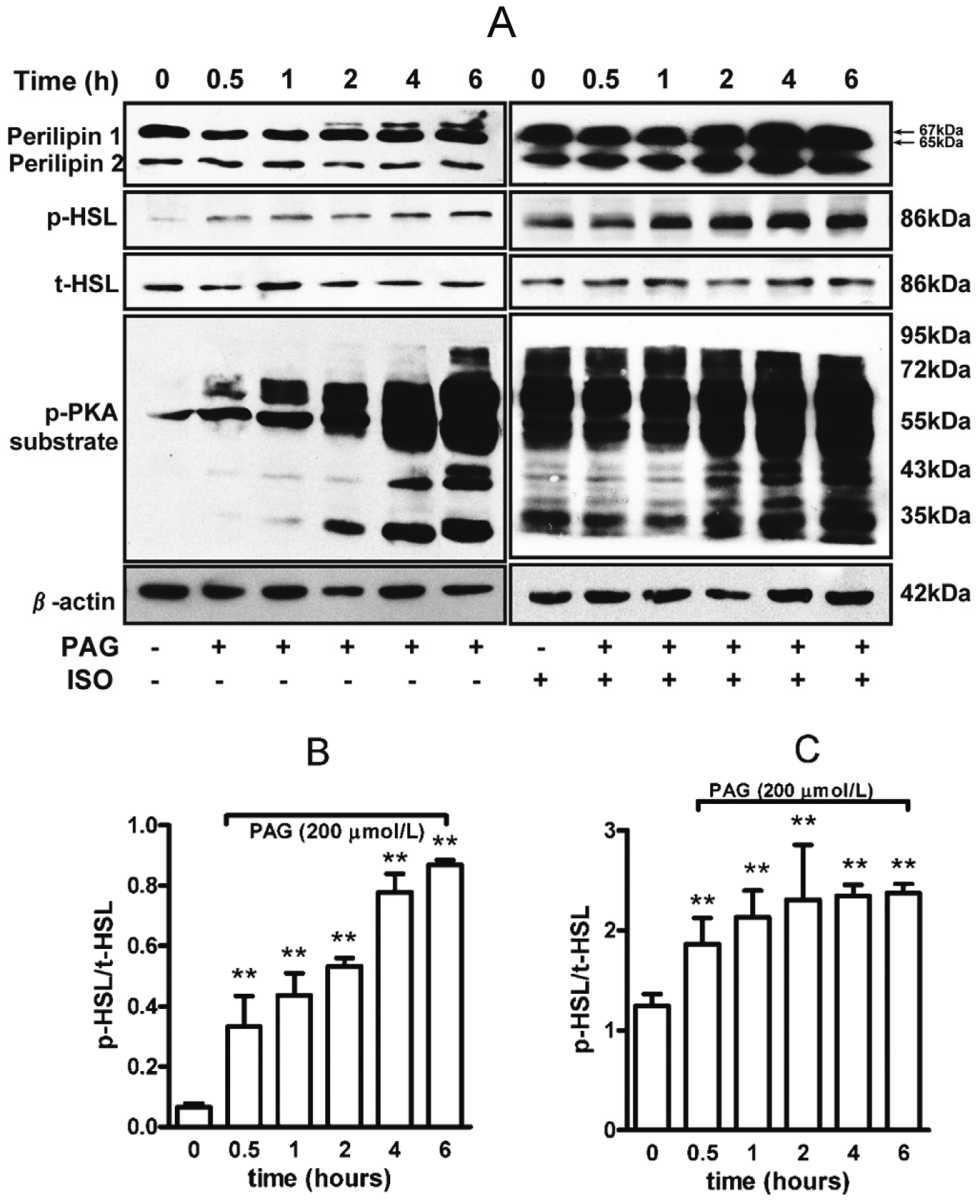
PAG increased phosphorylated PKA substrate, perilipin and hormone sensitive lipase (HSL) in rat adipocytes. (A) Lysates of adipocytes treated with 200 µmol/L PAG were separated by SDS-PAGE on low-Bis concentration gels and underwent immunoblot analysis with an anti-perilipin antibody. The band shift from 65 kDa (native) to 67 kDa (phosphorylated) perilipin 1 indicates hyperphosphorylation of full-length perilipin 1. The 46-kDa band is perilipin 2. Phosphorylated HSL at Ser^659^ and phosphorylated PKA (p-PKA) substrate was determined. Relative expression of p-HSL to total HSL was analyzed by band density under basal (B) or isoproterenol (1 µmol/L)-stimulated conditions (C). Data are mean ± SD. ** P<0.01 vs. untreated with PAG.

HSL is a key lipase to hydrolyze triglyceride in adipocyte. We found that PAG treatment time-dependently increased phosphorylated HSL at Ser^659^ (Fig. 2A and B) with or without isoproterenol (Fig. 2A and C). Activation of PKA phosphorylated perilipin 1 (a well-known lipid-droplet–associated protein [22]), facilitated HSL translocation to the surface of lipid droplet then accelerated triglyceride hydrolysis [23]. Here, we found that PAG time-dependently increased the phosphorylation of perilipin 1 and PKA substrate (Fig. 2A and B) under basal or isoproterenol-stimulated conditions (Fig. 2A), which suggested that PAG inhibition of endogenous H_2_S might elevate cellular cAMP and activate PKA, thereby stimulating the lipid catalytic reaction by a PKA-perilipin1/HSL pathway.

### H_2_S Precursor and H_2_S Chronic Release Donor Inhibited Lipolysis

**Figure 3:**
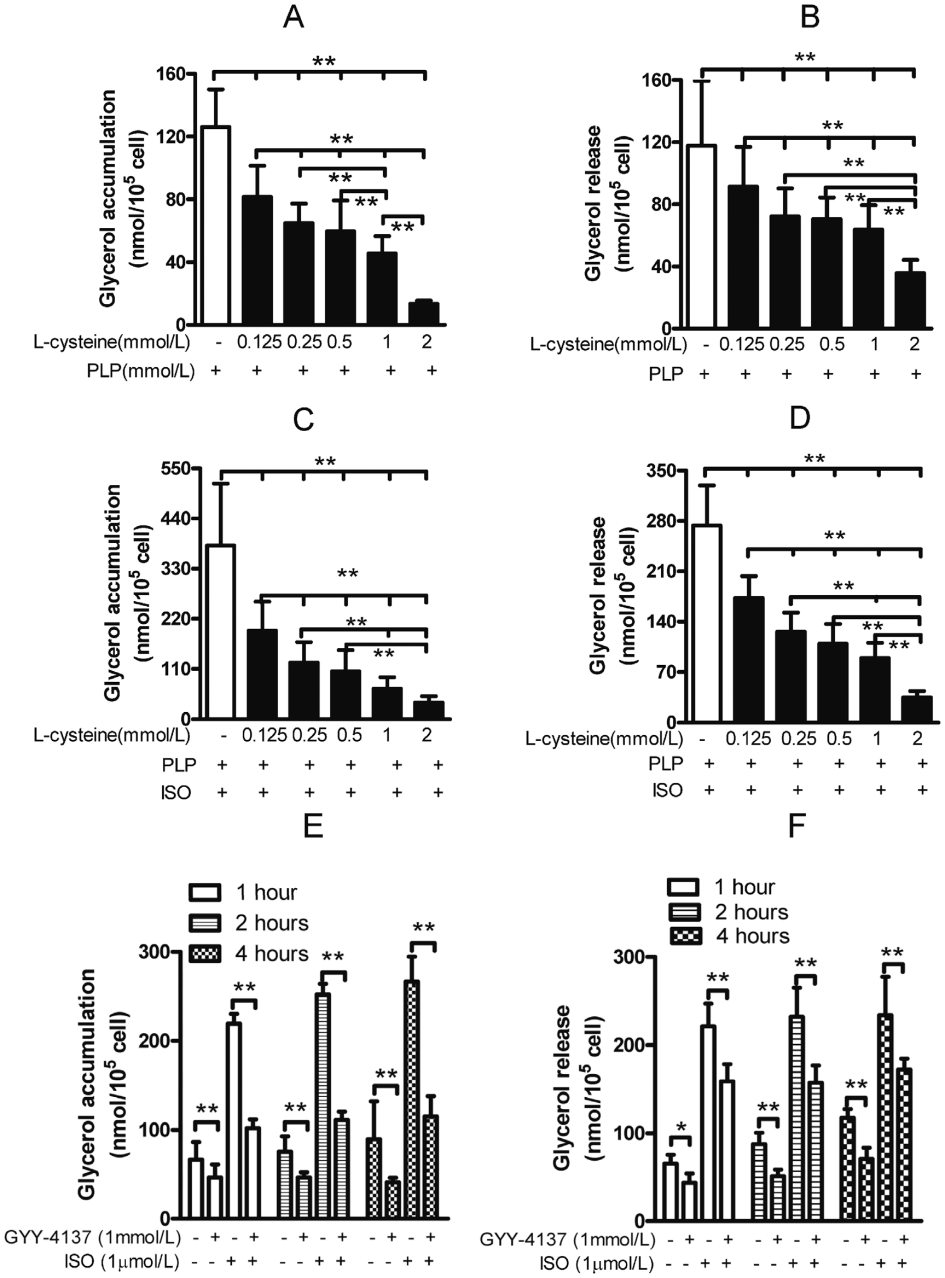
H_2_S precursor and donor inhibited lipolysis in rat adipocytes. Adipocytes were supplemented with L-cysteine plus pyridoxial phosphate (PLP) for 1 hr to increase endogenous H_2_S, then glycerol accumulation (A) or release in medium (B) was measured. The isoproterenol-stimulated glycerol accumulation (C) and release (D) was assayed with L-cysteine and pyridoxial phosphate treatment. After treatment with GYY-4137 (H_2_S release donor, 4–5 nmol/25 min then plateaued at least 75 min or more) for 2 hr, glycerol accumulation (E) or released (F) was measured under basal or isoproterenol-stimulated conditions. Data are mean ± SD from 10 experiments. * P<0.05; **P<0.01.

L-cysteine is a major precursor of endogenous H_2_S. As an H_2_S precursor, cysteine with CSE catalysis needs pyridoxial phosphate as a co-enzyme. Here we found that L-cysteine (from 125 µmol/L to 2 mmol/L) plus pyridoxal phosphate dose-dependently reduced basal (Fig. 3A and Fig. 3B) and isoproterenol-stimulated glycerol accumulation and glycerol release (Fig. 3C and Fig. 3D). To determine the direct effect of H_2_S, we treated adipocytes with GYY4137, a chronic H_2_S donor (H_2_S release, 4–5 nmol/25 min, then a plateau for at least 75 min [24]), for 2-h with or without isoproterenol and found that GYY4137 lowered the basal and isoproterenol-stimulated lipolysis (Fig. 3E and F). These data confirmed that L-cysteine endogenous release of H_2_S via CSE inhibited lipolysis in rat adipocytes.

**Figure 4:**
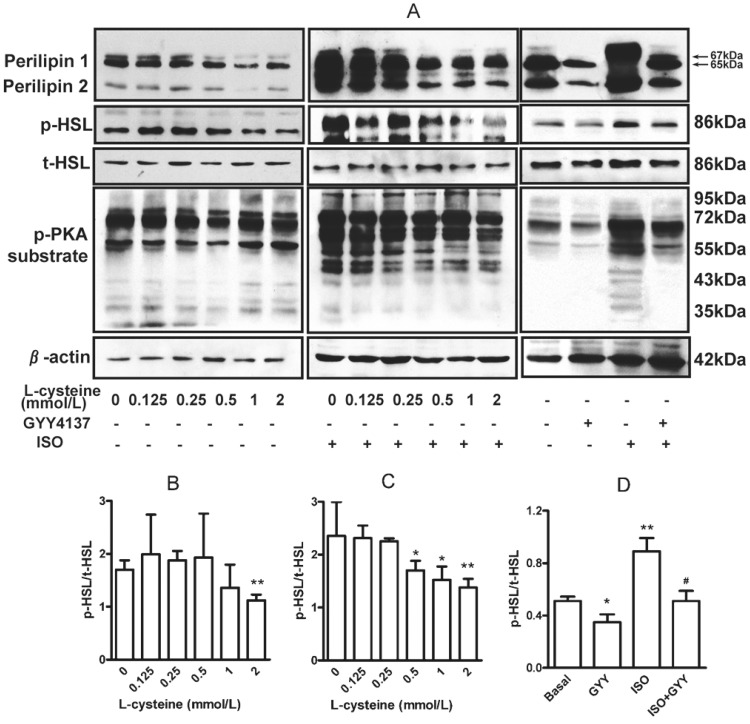
Endogenous H_2_S or chronic H_2_S donor reduced phosphorylated PKA substrate, perilipin 1 and HSL activity. (A) After treatment with L-cysteine plus pyridoxial phosphate or GYY4137, adipocyte lysates were separated by SDS-PAGE, and phosphorylated PKA substrate, perilipin 1 and HSL activity were assayed. The relative phosphorylated level of HSL to total HSL was compared after treatment with L-cysteine (B), isoproterenol (C) or GYY4137 (D).

H_2_S reduced isoproterenol-induced cAMP elevation and forskolin-stimulated adenylyl cyclase activity [25], then dose-dependently lowered cAMP-dependent PKA activation, as evidenced by decreased phosphorylation of the PKA substrate (Fig. 4A) under basal (Fig. 4B) and isoproterenol-stimulated conditions (Fig. 4C). H_2_S also inhibited phosphorylation of perilipin and HSL at Ser^659^ site (Fig. 4A, D) with or without isoproterenol stimulation, thus blocking HSL translocation to lipid droplet [8] for decreased lipolysis activity. These data suggest that the cAMP-PKA-perilipin/HSL pathway is involved in regulating the lipolysis by endogenous H_2_S in adipocytes.

### PAG and GYY4137 Regulated the Adipose Tissue Lipolysis In Vivo

**Figure 5:**
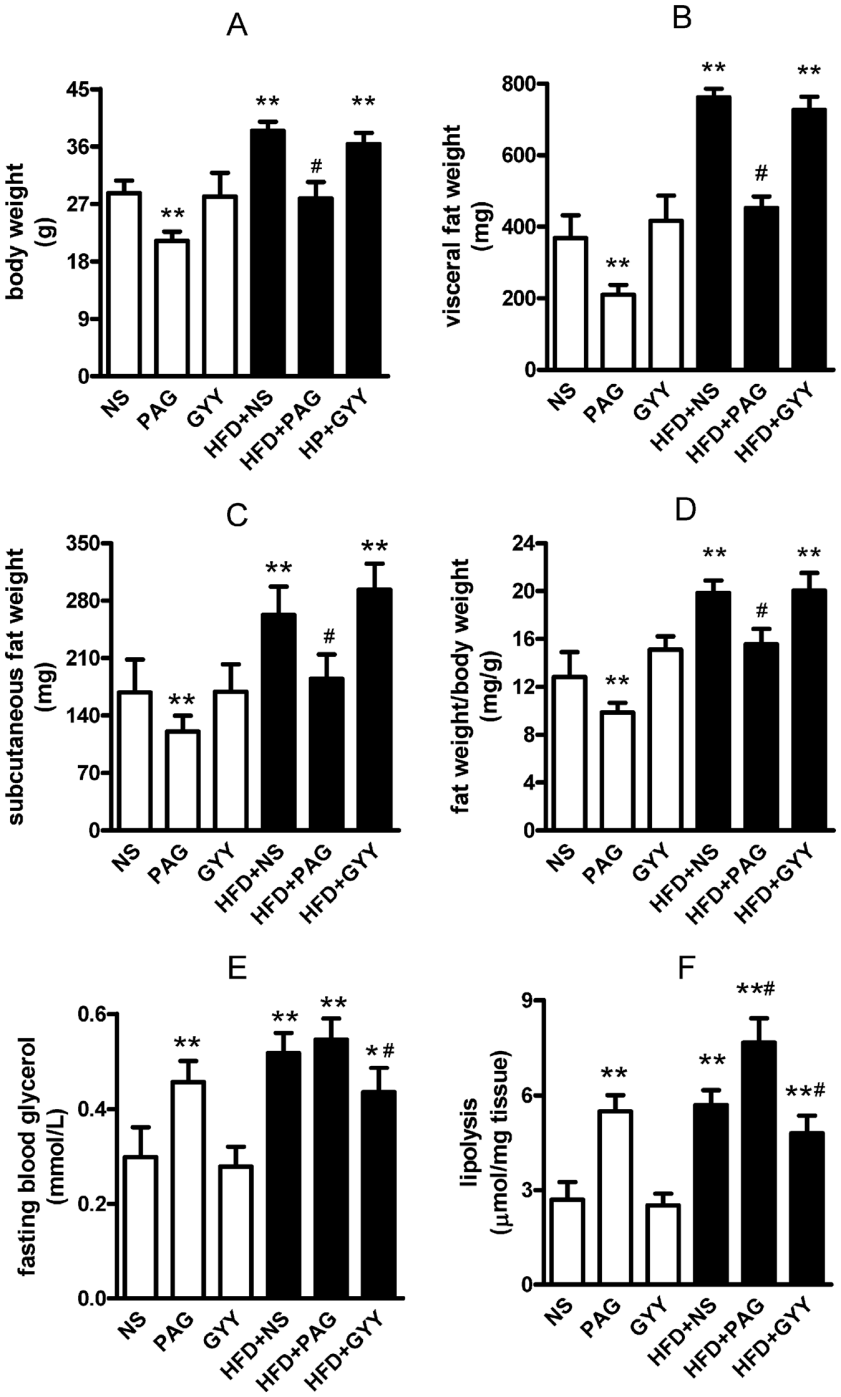
The basal characteristics changes and in vivo lipolysis after treatment by PAG and GYY4137 in HFD obesity mice. Obesity mice were induced by high fatty diet (45% energy from fat), normal diet (10% energy from fat) as control. PAG (30 mg/kg/day in saline) and GYY4137 (200 µmol/kg/day in saline) were administrated by subcutaneous injection and saline injection as control. After 13 weeks, body weight (A), visceral fat weight (B), subcutaneous fat weight (C) were measured then the fat weight/body weight (D) were counted. Fasting blood glucose (E) was assayed by glucometer. 20 mg epidymal adipose tissue from each mouse was incubated in serum-free and phenol red-free DMEM for 1-h, the release glycerol for evaluation lipolysis of mice (F) was assayed. All data are means ± SD. * P<0.05, ** P<0.01 versus control mice; # P<0.05 versus HFD saline injection mice.

Dysfunction of adipose lipolysis contributed to pathogenesis of insulin resistance in obesity. To investigate the role of adipose endogenous CSE/H_2_S in triglyceride lipolysis in vivo, we fed mice by HFD (45% energy from fat) for 13 weeks to induce obesity and normal diet (10% energy from fat) as control. As Fig. 5A–D shown, HFD significantly increased C57BL/6J body weight (Fig. 5A, P<0.01), visceral fat weight (including epididymal fat pad, perinephric fat and retroperitoneal fat, Fig. 5B, P<0.01) and subcutaneous fat weight (Fig. 5C, P<0.01), resulting in increasing ratio of fat weight/body weight (Fig. 5D, P<0.01). Association with fat mass increased by HFD, CSE protein expression and endogenous H_2_S production decreased in adipose tissues (Figure S1 in File S1). PAG inhibited CSE expression and H_2_S production (Figure S1 in File S1), lowered the basal and HFD induced body weight growth (Fig. 5A, P<0.01) and blunted the fat mass increase (evidenced by visceral, subcutaneous fat weight and ratio of fat weight/body weight, Fig. 5B–D, all P<0.01). Leptin is a marker of obesity by HFD. Here we found that PAG antagonized the high plasma leptin level induced by HFD (Figure S2 in File S1), which was according to reduction of fat mass. H_2_S donor (GYY4137) did not affect these basal physiological characteristics. Triglyceride lipolysis release 1 glycerol and 3 free fatty acid. Circulatory free fatty acid was quickly lowered by uptake, oxidation or reesterfication in tissue. So we used circulatory glycerol to assess the adipose lipolysis in vivo. As Fig. 5E shown, PAG treatment increased fasting blood glycerol in normal chow mice (P<0.01), slightly (but not statistical significant) increased it in HFD mice. GYY4137 lowered fasting blood glycerol level in HFD mice (Fig. 5E, P<0.01) which suggested that H_2_S lowered basal lipolysis in obese fat. To confirm the effects of PAG and GYY4137, we measured the direct lipolysis in isolated adipose tissues and found that PAG-treatment increased glycerol release from adipose tissues in both control and HFD mice; GYY-treatment reduced lipolysis in HFD adipose tissues (Fig. 5F, P<0.01). Food uptake is an important factor of obesity, so we measured the food consumption and found that PAG and GYY4137 treatment did not affect the food consumption (FigureS3 in File S1). These data implied that PAG continuously elevated lipolysis blunted HFD induced obesity; H_2_S donor seemly lowered the lipolysis in obese adipose, but did not accelerate fat mass deposition.

### Both Increase and Decrease CSE/H_2_S Reduced Global Insulin Resistance In Vivo

**Figure 6:**
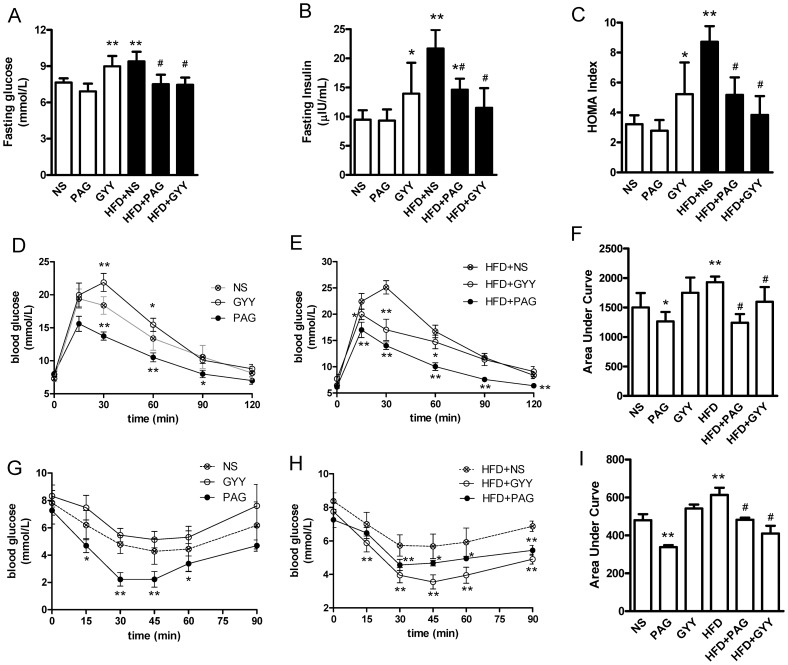
The effects of PAG and GYY4137 on insulin sensitivity in HFD mice. For assessing insulin sensitivity, fasting blood glucose (A), fasting serum insulin level (B) and HOMA index (C) was counted. After fasting 16-hrs, oral glucose tolerance test (OGTT) in normal chow mice (D) and HFD mice (E), area under curve for OGTT (F); insulin tolerance test (ITT) in control mice (G) and HFD mice (H) and area under curve for ITT (I) were assayed. All data are means ± SD. * P<0.05, ** P<0.01 versus control mice; # P<0.05 versus HFD saline injection mice.

To investigate the role of endogenous CSE/H_2_S in insulin resistance, we treated mice with PAG or GYY4137 for 13 week, then we assessed the insulin sensitivity. Here, we found that PAG per se did not affect fasting blood glucose, fasting blood insulin and HOMA index (Fig. 6A–C); slightly increased insulin sensitivity evidenced by decrease OGTT curve area (Fig. 6D and F) and increase glucose response to insulin (ITT assay, Fig. 6G and I) in normal chow mice. Interestingly, PAG significantly lowered insulin resistance in HFD obese mice evidenced by decrease fasting blood glucose and insulin, HOMA index (Fig. 6A–C, P<0.01), OGTT curve area (Fig. 6E and F, P<0.01) and ITT curve area (Fig. 6H and I, P<0.01).

In normal chow mice, H_2_S donor-GYY4137 slightly increased blood glucose and insulin levels for fasting 6-h (Fig. 6A and B, P<0.05), resulting in elevation of HOMA index (Fig. 6C, P<0.05) comparison to saline injection mice. However, GYY4137 lowered these parameters in HFD mice (Fig. 6A–C, P<0.05). In normal chow mice, GYY4137 delayed the blood glucose peak in OGTT curve (Fig. 6C), and slightly decreased the glucose response to insulin (Fig. 6F). In HFD obese mice, GYY4137 lowered the OGTT curve area (Fig. 6E and F) and increased the effects of lowering blood glucose by insulin (Fig. 6H and I). These data suggested that blocked endogenous CSE enzyme activity lowered fat mass growth association with reduction insulin resistance in HFD obese mice. More interestingly, H_2_S donor also reduced insulin resistance in HFD mice, but slightly decreased insulin sensitivity in normal chow mice. The bilateral regulation of CSE/H_2_S in insulin resistance also implied that different pathway or signals were involved in H_2_S regulation in physiological and/or pathological condition.

**Figure 7:**
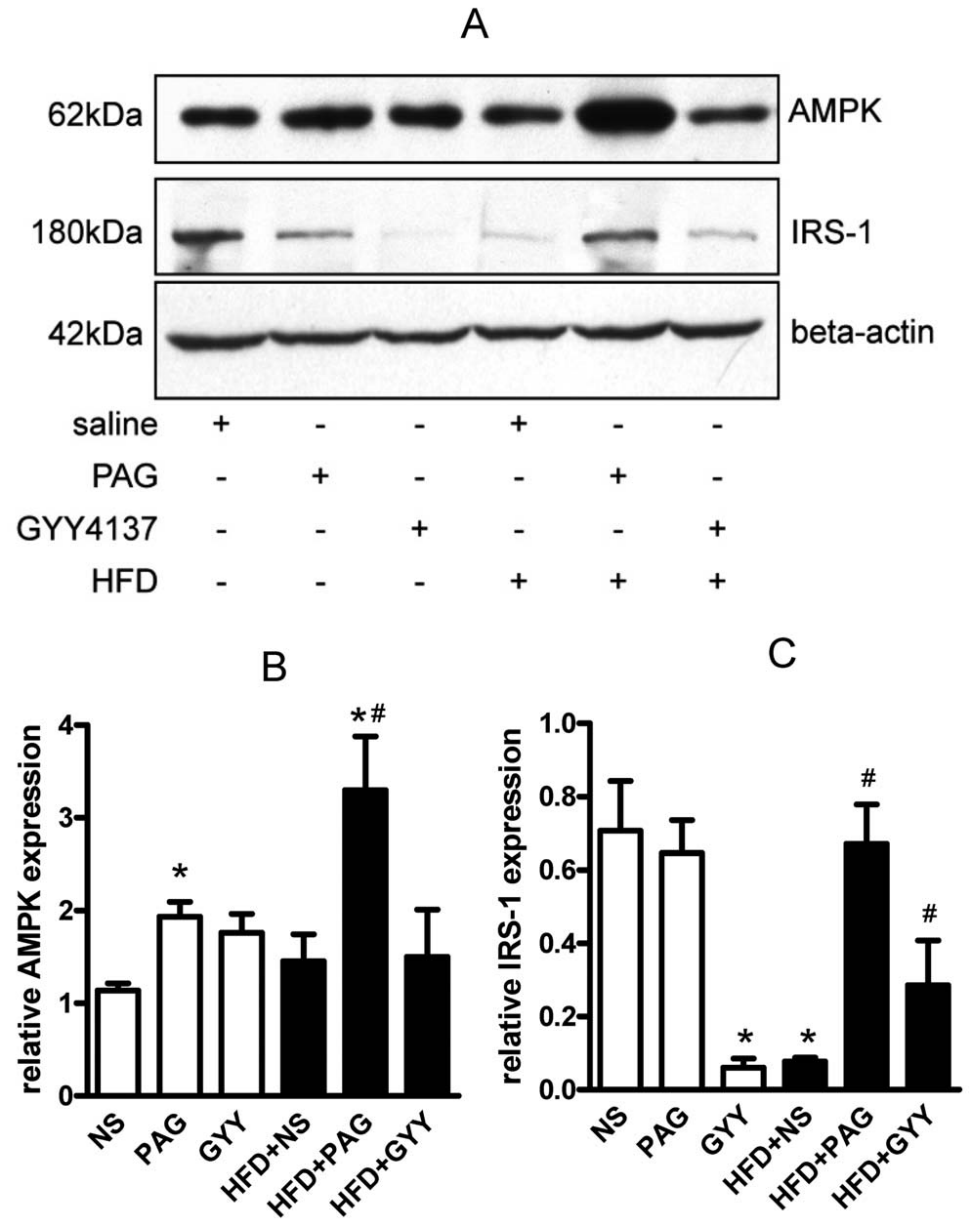
Alterations of AMPK and IRS-1 protein expression in epidymal adipose tissues. Relative protein expression of AMPK and IRS-1 in adipose were measured by western blot (A). Gray analysis was performed for quantization of AMPK (B) and IRS-1 (C). Six independent experiments were performed. All data are means ± SD. * P<0.05 versus control mice; # P<0.05 versus HFD saline injection mice.

AMPK is an energy sensor and play an essential role in insulin signal. AMPK directly phosphorylated IRS-1 then increased insulin sensitivity. Here, we found that PAG up-regulated AMPK protein (Fig. 7A and B, P<0.05) in adipose tissues of control mice and HFD mice, but GYY4137 did not. PAG also increased IRS-1 protein expression in HFD mice. These findings suggested that AMPK-IRS-1 pathway may be involved in the regulation of PAG to antagonize adipose tissues insulin resistance. Giving GYY4137 treatment, lowered adipose IRS-1 protein expression in control mice but up-regulated it in HFD mice (Fig7.A and C). In present study, PAG and GYY4137 were systemic administration, so we also measured the AMPK and IRS-1 expression in skeletal muscle. As supplemental data Fig. S4 (Figure S4 in File S1)shown, both PAG and GYY4137 up-regulated AMPK and IRS-1protein expression in HFD mice; PAG also increased IRS-1 protein expression in normal chow mice. These results might explain the bilateral regulation of H_2_S in insulin sensitivity in vivo.

## Discussion

Dysfunction of lipolysis contributed to pathogenesis of insulin resistance. In present study, we found that inhibition of adipocyte endogenous CSE/H_2_S with PAG increased basal and isoproterenol stimulated lipolysis, oppositely H_2_S donor (GYY4137) inhibited them and PKA-HSL/perilipin pathway involved in the regulation. PAG increased blood glycerol and adipose lipolysis but not lowered food uptake, which thus blunted HFD induced obesity, and reduced insulin resistance from HFD mice; GYY4137 did not change HFD induced fat mass increase, but ameliorated the insulin resistance in obese mice.

H_2_S is a metabolic production source from cysteine dependent on CSE in adipose tissues. Several clinical studies have reported a positive association of total cysteine level (including cysteine, reduced cysteine, cystine, and mixed disulphides) with body mass index (BMI) [26]–[30]; with fat mass (as measured by dual energy X-ray absorptiometry) contributing the most to the BMI [31]. CSE-knockout mice showed lower plasma cysteine and H_2_S levels, and lower body weight, of which white adipose tissue mass (34% of wild-type) was the most contribution to the body weight lost [32]. These studies strongly suggest that cysteine/CSE contributes to functional regulation of adipocytes. Here we found that CSE inhibitor-PAG induced robust basal and isoproterenol stimulated lipolysis; H_2_S precursor-L-Cysteine or donor-GYY4137 (a water soluble, stable, chronic releasing H_2_S donor [24]) inhibited them. In normal chow and HFD mice, PAG also increased lipolysis evidenced by elevated serum glycerol and lipolysis reaction in isolated adipose tissues, but did not affect food consumption, then, blunting fat mass deposition and body weight increase. H_2_S donor lowered lipolysis in vivo in HFD mice but not in normal chow mice. GYY4137 just offer about 4–5 nmol per 25 min [24], and accumulated in liver, kidney and other organ during 2 hours after bonus injection [33]. Thus systemic administration GYY4137 just partly inhibited lipolysis in short times, which partly explained that GYY4137 treatment did not increase fat mass and body weight. These findings supported that adipose endogenous CSE/H_2_S system contributed to lipolysis reaction, which might be a reason of high serum blood cysteine positive regression with BMI and body fatty mass [31].

H_2_S attenuated catecholamine-induced cellular cAMP elevation [25], inhibited PKA activation which reduced phosphorylated HSL and perilipin 1, thus blocked HSL translocation to lipid droplet [1] resulting in lowered triglyceride lipolysis. The hypothesis is evidenced by the phosphorylation of PKA substrate, HSL at ser^659^ site, perilipin 1, and cysteine activated by PAG or inhibited by GYY4137 (Fig. 4). These findings suggested that PKA-HSL/perilipin 1 pathway is involved in the lipolytic regulation by H_2_S. Plasma cysteine releases H_2_O_2_ via Cu^2+^-dependent auto-oxidation [34], [35]. H_2_O_2_ also inhibits hormone-sensitive lipase activity by forming an intersubunit disulfide bond within cAMP-dependent PKA [36], which might be a molecular mechanism of cysteine action in the lipolysis response *in vivo*. In isolated adipocytes, Cu^2+^ absence limited this response model. Antioxidants such as N-acetyl-L-cysteine or diphenyleneiodonium lowered the adenylyl cyclase activity by increasing Gi protein expression, then decreasing intracellular cAMP level [37]. H_2_S is a strong antioxidant [38]; whether H_2_S upregulates receptor-dependent or -independent Gi protein expression or function causing inhibition adenylyl cyclase activity needs further investigation. Global CSE-knockout mice showed lower fat weight [32], which might be caused by increasing adipose lipolysis because of CSE deficient. Unfortunately, authors did not measure adipocyte lipolysis activity in CSE-knockout mice [32].

Obesity is an independent risk factor of diabetes. In adipocyte of HFD obese mice, basal lipolysis is increased but catecholamine-stimulated lipolysis is blunted [39]. Saturated fat in the high fatty diet increased adipose TNF-α expression and macrophage infiltration [40], these local inflammatory cytokine impaired insulin effects on glucose uptake activity and lipolysis [41]. PAG inhibited endogenous CSE/H_2_S system and dose-dependently recovered the glucose consumption and uptake which impaired by TNF-α in adipocyte [42]. PAG also up-regulated AMPKα (an important kinase involved in insulin sensitivity) and IRS-1 protein (insulin signal transduction) in adipose and skeletal muscle, which means blocking CSE/H_2_S stimulated adipose energy output similar to energy deprivation. These data suggested PAG ameliorated adipose insulin resistance partly by reduction adipose inflammation and activated AMPK pathway.

H_2_S donor inhibited the basal lipolysis in obese adipose tissues, increased insulin resistance in normal chow mice, but decreased that in HFD mice. H_2_S per se inhibited rat mature adipocyte basal glucose uptake and insulin stimulated glucose uptake [12], which may contribute to increase insulin resistance in normal chow. Whereas, H_2_S donor antagonized high glucose lowered phosphorylated Akt, phospho-IRS-1 and type four glucose transporter (GLUT4) protein expression by inhibition PTEN and NF-κB activation [43]. Another work also found that CSE involved in the protective effects of vitamin D on insulin resistance by high glucose [14]. The present study also found that H_2_S donor increase IRS-1 protein expression in obese adipose and skeletal muscle. These data suggested that CSE/H_2_S system might regulate gene transcription of glucose metabolic enzyme or transporter protein by nuclear receptor such as vitamin D receptor. The bilateral regulation of CSE/H_2_S system in glucose metabolism suggested that CSE/H_2_S might act as an energy balancer. In physiological condition, CSE/H_2_S system is apt to reduce energy consumption thus slightly decrease glucose utilization; while under stress or inflammation, CSE/H_2_S antagonize injury and increase glucose utilization resulting in increasing insulin sensitivity.

In conclusion, the endogenous CSE/H_2_S system in adipocytes regulated lipolysis by PKA-perilin/HSL pathway. Inhibition of CSE/H_2_S induced robust lipolysis thus blunted adipose increase and lowered insulin resistance by AMPK pathway. Although H_2_S donor lowered lipolysis, it ameliorated insulin resistance by up-regulating IRS-1 protein. The paradoxical regulation of CSE/H_2_S system in insulin sensitivity implied that CSE and H_2_S might have independent regulation mechanism and differential signal transduction. Clearance the regulation of CSE/H_2_S in energy metabolism may be helpful to understand the complicated interactive linkage of glucose, fat and sulfur containing amino acid in physiology and diseases.

## Supporting Information

File S1 **File containing all supporting information figures.** Figure S1: Changes of endogenous CSE/H_2_S system in epididymal adipose tissues. (A): CSE protein expression was measured by western blot. (B): Relative quantitative of CSE protein expression was analyzed by gray density of CSE and β-actin band. (C): Endogenous H2S production in adipose tissue was assayed by the methylene blue method. All data are means ± SD. * P<0.05, ** P<0.01 versus normal chow mice; # P<0.05 versus HFD mice. Figure S2: Alterations of plasma leptin measured by ELISA assay (ELISA kit from R&D Minneapolis, MN). All data are means ± SD. ** P<0.01 versus normal chow mice; # P<0.05 versus HFD mice. Figure S3: Food consumption was measured every 3 days. Figure S4: Alterations of AMPK and IRS-1 protein expression in skeletal muscle tissues. Relative protein expression of AMPK and IRS-1 in skeletal muscle were measured by western blot (A). Gray analysis was performed for quantization of AMPK (B) and IRS-1 (C). Six independent experiments were performed. All data are means ± SD. * P<0.05 versus normal chow mice; # P<0.05 versus HFD mice.
(DOC)
