# Supplementary figures and images for: Increase or Decrease Hydrogen Sulfide Exert Opposite Lipolysis, but Reduce Global Insulin Resistance in High Fatty Diet Induced Obese Mice
Source: PLoS One. 2013 Sep 13;8(9):e73892. doi: 10.1371/journal.pone.0073892 (PMC3772803; doi:10.1371/journal.pone.0073892)

Figure S1

A


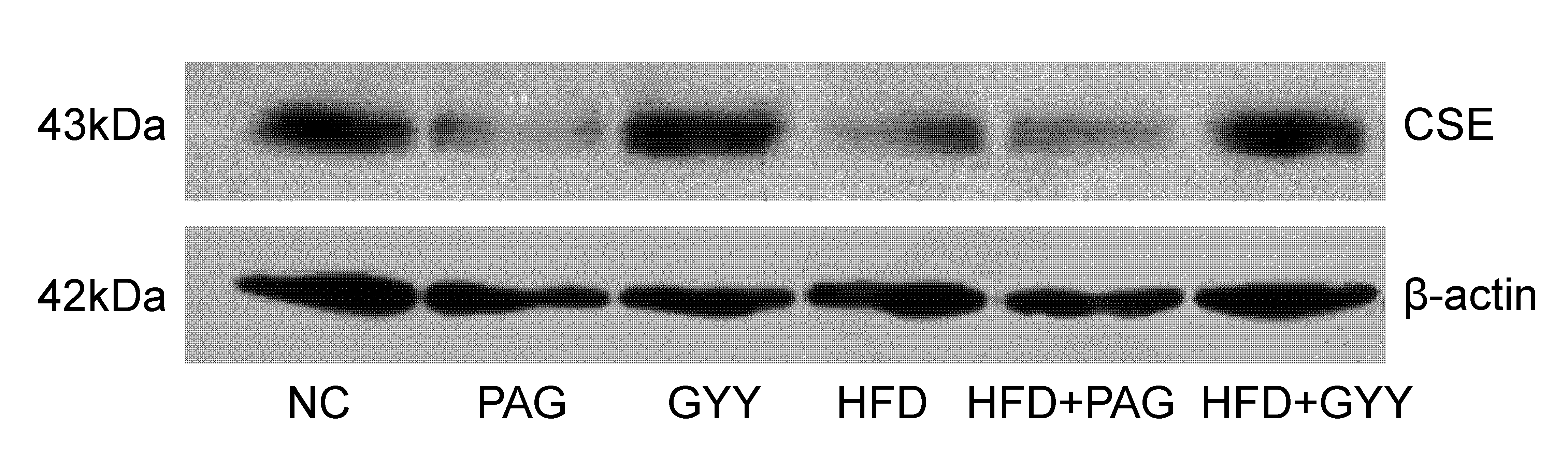


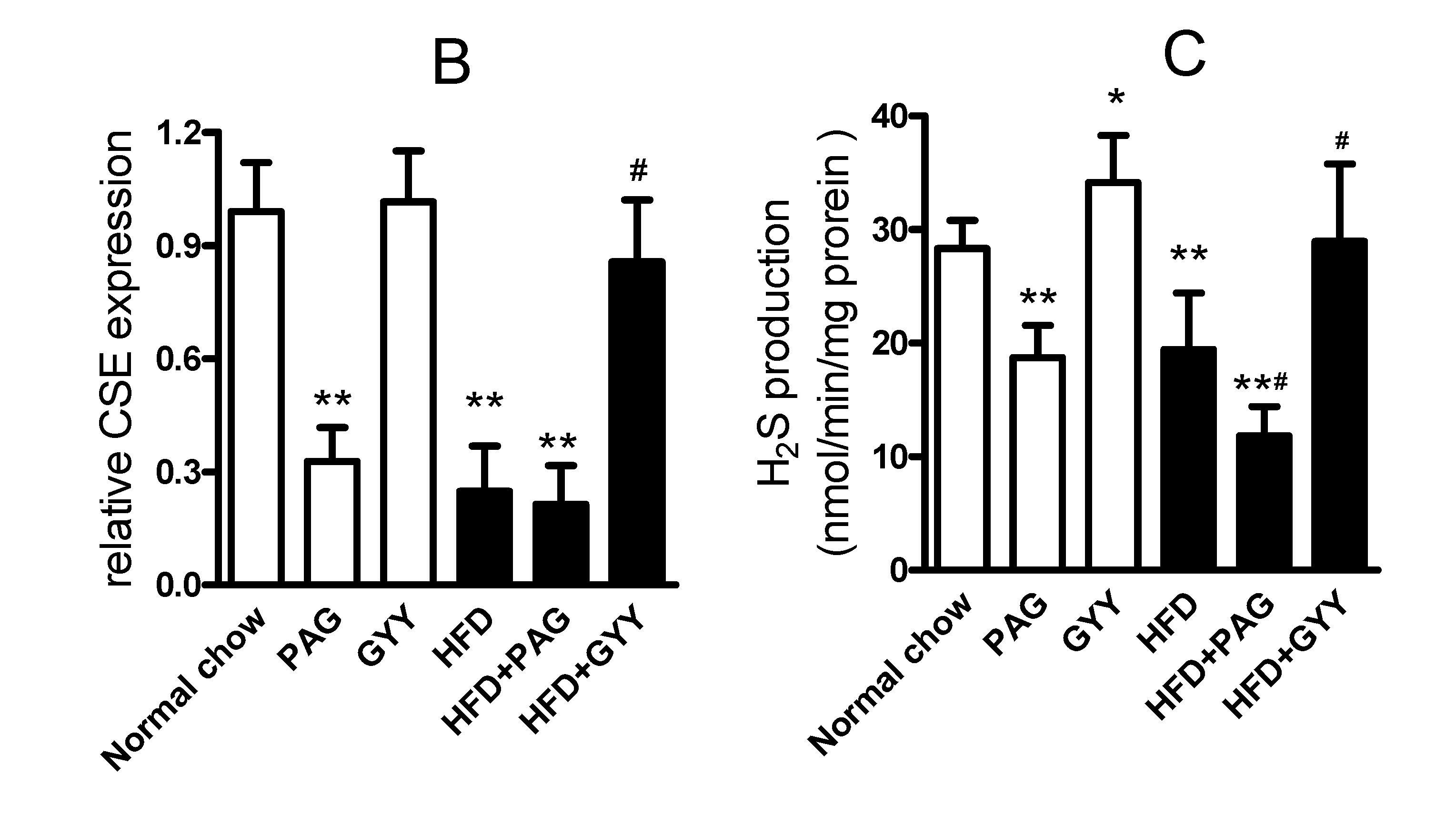


Figure S2

Figure S3


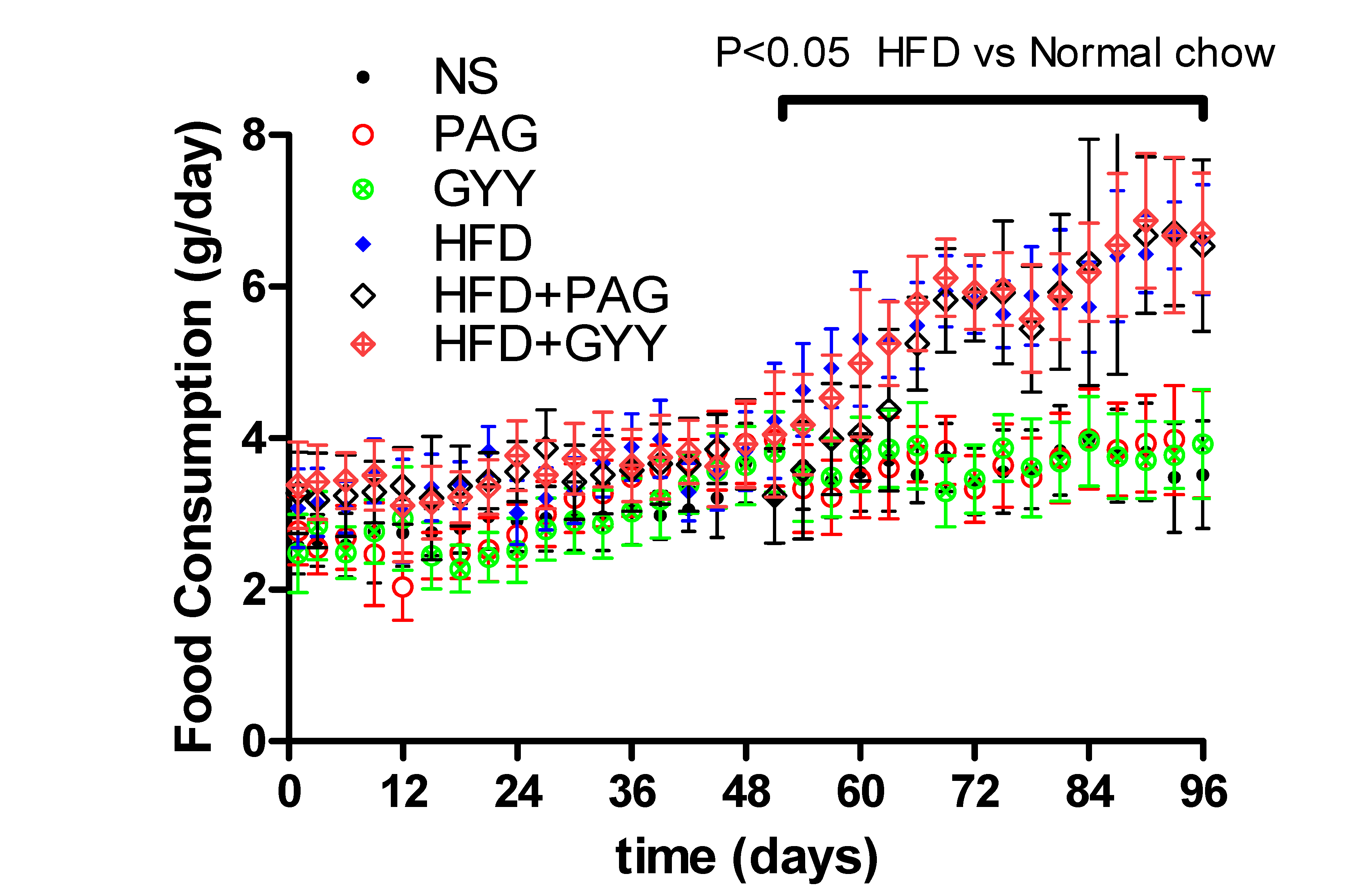


Figure S4


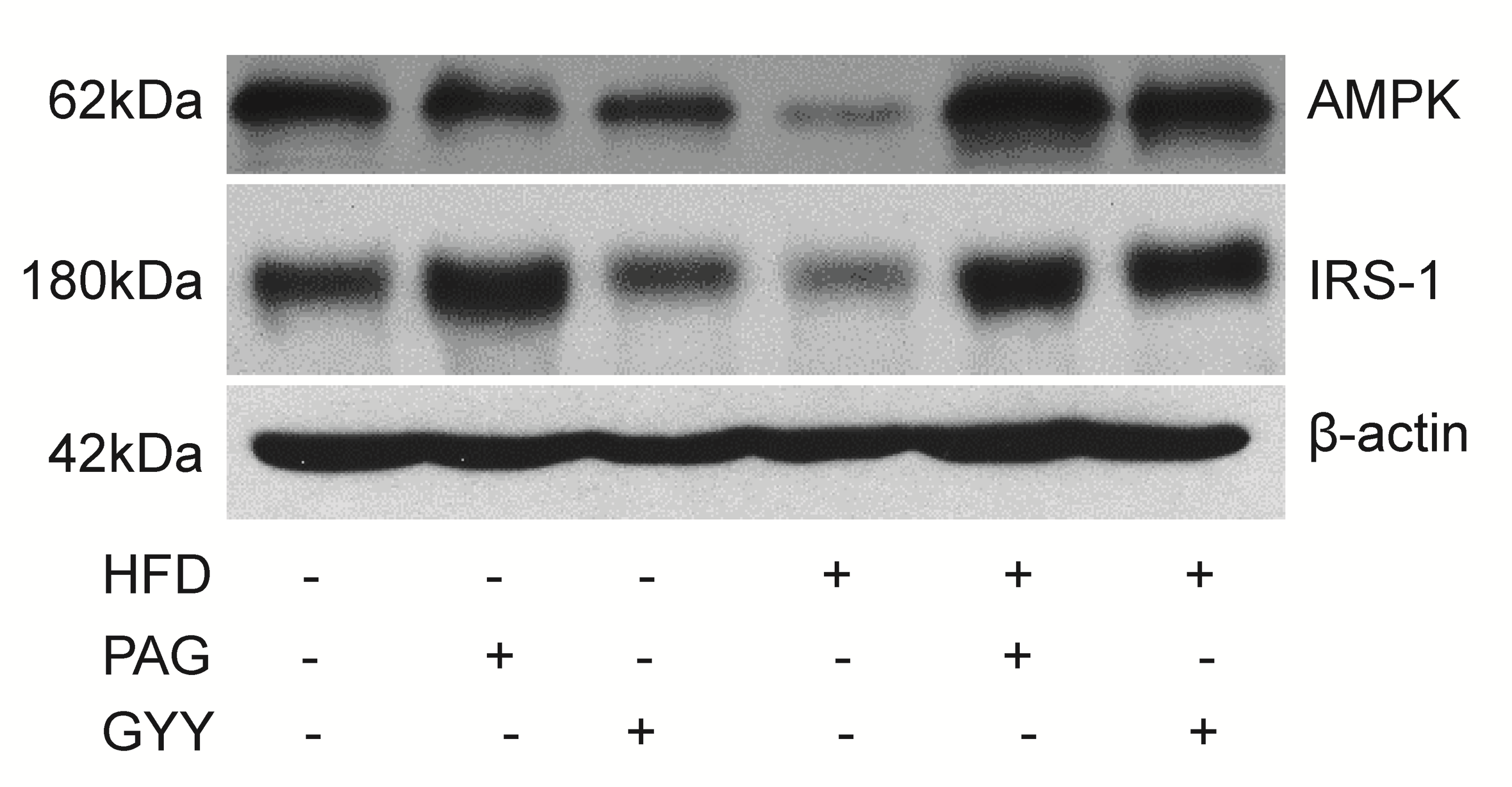


A


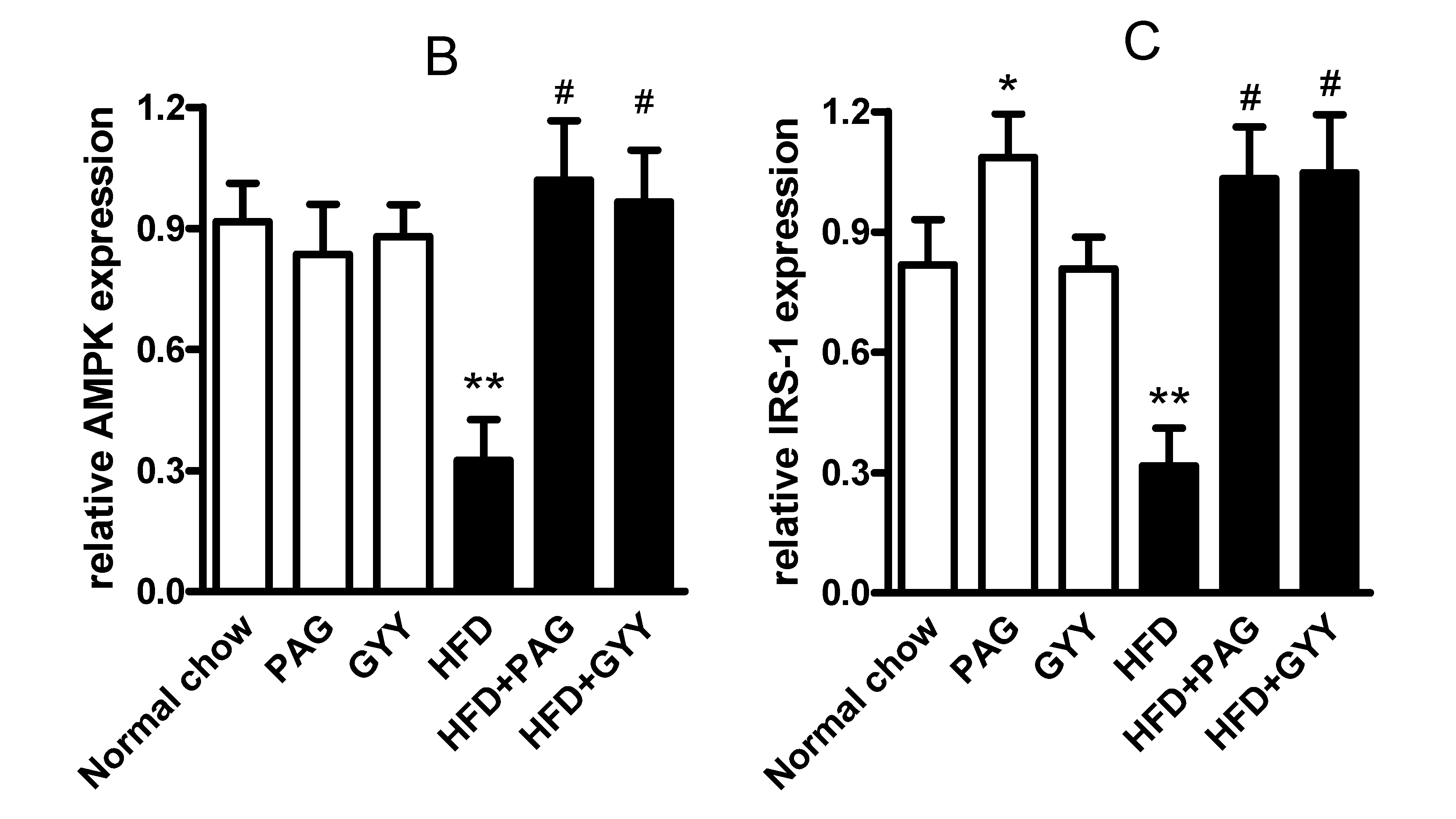

Supplement: File S1 — File containing all supporting information figures. Figure S1: Changes of endogenous CSE/H2S system in epididymal adipose tissues. (A): CSE protein expression was measured by western blot. (B): Relative quantitative of CSE protein expression was analyzed by gray density of CSE and β-actin band. (C): Endogenous H2S production in adipose tissue was assayed by the methylene blue method. All data are means ± SD. * P<0.05, ** P<0.01 versus normal chow mice; # P<0.05 versus HFD mice. Figure S2: Alterations of plasma leptin measured by ELISA assay (ELISA kit from R&D Minneapolis, MN). All data are means ± SD. ** P<0.01 versus normal chow mice; # P<0.05 versus HFD mice. Figure S3: Food consumption was measured every 3 days. Figure S4: Alterations of AMPK and IRS-1 protein expression in skeletal muscle tissues. Relative protein expression of AMPK and IRS-1 in skeletal muscle were measured by western blot (A). Gray analysis was performed for quantization of AMPK (B) and IRS-1 (C). Six independent experiments were performed. All data are means ± SD. * P<0.05 versus normal chow mice; # P<0.05 versus HFD mice. (DOC) [file pone.0073892.s001.doc]
